# Blocking oestradiol synthesis pathways with potent and selective coumarin derivatives

**DOI:** 10.1080/14756366.2018.1452919

**Published:** 2018-04-05

**Authors:** Sanna Niinivehmas, Pekka A. Postila, Sanna Rauhamäki, Elangovan Manivannan, Sami Kortet, Mira Ahinko, Pasi Huuskonen, Niina Nyberg, Pasi Koskimies, Sakari Lätti, Elina Multamäki, Risto O. Juvonen, Hannu Raunio, Markku Pasanen, Juhani Huuskonen, Olli T. Pentikäinen

**Affiliations:** a Department of Biological and Environmental Science and Nanoscience Center, University of Jyvaskyla, Jyvaskyla, Finland;; b School of Pharmacy, Devi Ahilya University, Indore, India;; c Department of Chemistry and Nanoscience Center, University of Jyvaskyla, Jyvaskyla, Finland;; d School of Pharmacy, University of Eastern Finland, Kuopio, Finland;; e Forendo Pharma Ltd, Turku, Finland;; f Institute of Biomedicine, University of Turku, Turku, Finland

**Keywords:** 3-Phenylcoumarin, 17-β-hydroxysteroid dehydrogenase 1 (HSD1), 3-imidazolecoumarin, aromatase, structure-activity relationship (SAR)

## Abstract

A comprehensive set of 3-phenylcoumarin analogues with polar substituents was synthesised for blocking oestradiol synthesis by 17-β-hydroxysteroid dehydrogenase 1 (HSD1) in the latter part of the sulphatase pathway. Five analogues produced ≥62% HSD1 inhibition at 5 µM and, furthermore, three of them produced ≥68% inhibition at 1 µM. A docking-based structure-activity relationship analysis was done to determine the molecular basis of the inhibition and the cross-reactivity of the analogues was tested against oestrogen receptor, aromatase, cytochrome P450 1A2, and monoamine oxidases. Most of the analogues are only modestly active with 17-β-hydroxysteroid dehydrogenase 2 – a requirement for lowering effective oestradiol levels *in vivo*. Moreover, the analysis led to the synthesis and discovery of 3-imidazolecoumarin as a potent aromatase inhibitor. In short, coumarin core can be tailored with specific ring and polar moiety substitutions to block either the sulphatase pathway or the aromatase pathway for treating breast cancer and endometriosis.

## Introduction

Despite the recent advances made in early tumour detection, clinical treatments and avoidance of menopausal hormone therapies, breast cancer continues to be the most common invasive cancer, and a second leading cause of cancer death for women^1^. Therefore, potent and selective pharmaceutical agents are actively sought to supplement and/or replace the often-invasive treatments and to lower the medical costs for all breast cancer patients.

A clear majority of breast cancer tumours are oestrogen receptor (ER) positive. The tumour growth is linked to high ER numbers and/or their increased activity due to high 17-β-oestradiol (E_2_) levels. Hence, the existing drugs generally aim to block the ER function in breast tissue or limit its function indirectly by lowering the E_2_ production. The aromatase pathway produces E_2_ from androgen hormones whereas the sulphatase pathway converts oestrone sulphate (E_1_S) into oestrone (E_1_) and ultimately to E_2_. Although the aromatase pathway (active in local E_2_ production) is in a lesser role with most breast cancers^2^, widely used drugs, such as anastrozole focus on blocking it instead of the more prominent sulphatase pathway.

**Figure 1.**
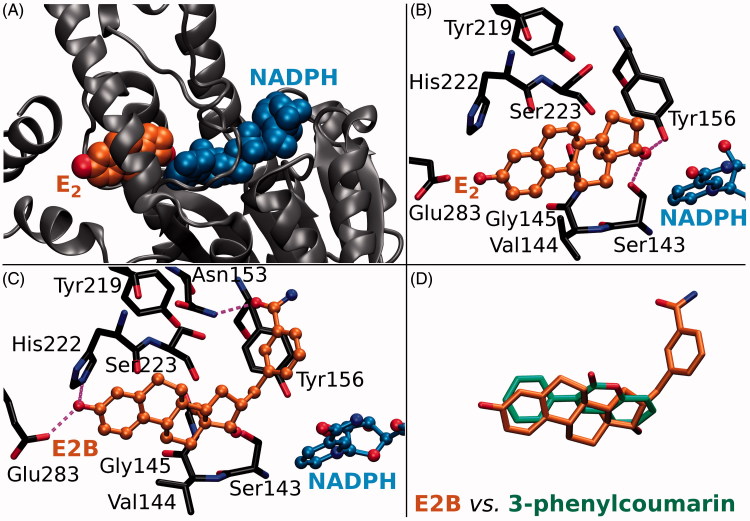
The ligand binding at the active site of 17-β-hydroxysteroid dehydrogenase 1. (A) Oestradiol (E_2_; orange backbone) and oxidised cofactor nicotinamide adenine dinucleotide phosphate (NADP; blue) are shown as CPK models in complex with the HSD1 structure (grey cartoon; PDB: 1A27). (B) The H-bonding between E_2_ (ball-and-stick models with orange backbone) and the residues lining the active site (stick model with black backbone) are shown with magenta dotted lines. The substrate oestrone (E_1_) acquires a proton (or H^+^) from NADPH, the reduced form of the cofactor, via the hydroxyl group of Tyr156 (E_2_ + NADP^+^ + ⇌ E_1_ + NADPH), which is H-bonding with the 17-keto group of the reaction product E_2_. (C) Inhibitor E2B (ball-and-stick model with orange backbone; PDB: 3HB5)^25^ binding at the HSD1 active site blocks E_2_ binding (B vs. C). (D) The 3-phenyl and coumarin rings of the docked analogues (stick model with green backbone) align in a roughly similar manner inside the active site as the steroid ring of E2B (stick model with orange backbone).

17-β-hydroxysteroid dehydrogenase 1 (HSD1 or 17-β-HSD1; Figure 1(A)) has a crucial role in the final steps of E_2_ biosynthesis via the sulphatase pathway. HSD1 homodimer reduces the C17-keto group of E_1_ by acquiring a proton (H^+^) from the cofactor nicotinamide adenine dinucleotide phosphate (NADPH) to produce E_2_ (Figure 1(A,B)). In contrary, 17-β-hydroxysteroid dehydrogenase 2 (HSD2 or 17-β-HSD2) promotes the oxidation of the C17-hydroxyl group on E_2_ by donating H^+^ to the cofactor to produce E_1_. HSD1 overexpression is a strong signal for breast cancer – present in ∼50% of breast tumours – and, furthermore, HSD2 is known to have an inhibitory effect in the breast tumourigenesis^3^^,^^4^. HSD1 is also linked to other cancer types, such as gastric^5^ and cervical cancer^6^, and, additionally, in endometriosis elevated E_2_ production is promoted by increased HSD1 and, inversely, lowered HSD2 expression^7^.

**Figure 2.**
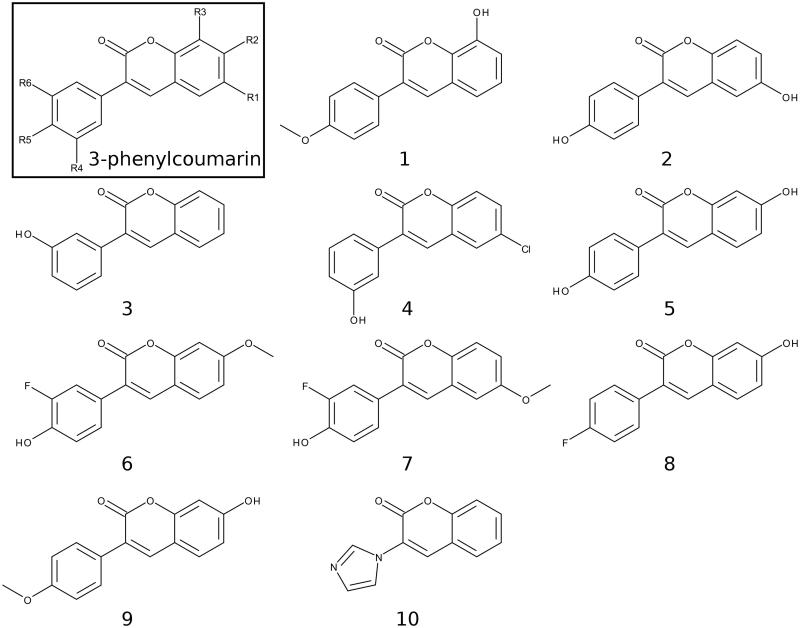
2D structures of the coumarin derivatives. The 3-phenylcoumarin analogues **1–7** produce HSD1 inhibition at a varying degree, but **8** and **9** were found to be inactive (Table 1). Compound **10** or 3-imidazolecoumarin inhibit aromatase instead of HSD1.

A vast number of steroidal (^8–10^; e.g. E2B in Figure 1(C)) and non-steroidal (see e.g.^11–13^) compounds are known to inhibit the HSD1 activity, but none of these promising leads has passed clinical trials so far. There are also several X-ray crystal structures of HSD1 in both ligand-free, substrate-, and inhibitor-bound states to facilitate rational structure-based drug discovery. Here, 3-phenylcoumarin (or 3-arylcoumarin) is shown to be a suitable non-steroidal scaffold for building small-molecule inhibitors targeting HSD1 (Figure 2; Table 1).

**Table 1.** Inhibitory profiles of the 3-substituted coumarin compounds.

#	HSD1 inhibition at 100 nM^a^ (%)	HSD1 inhibition at 1 µM^b^ (%)	HSD1 inhibition at 5 µM (%)	HSD1 inhibition ∼ pIC_50_	HSD2 inhibition at 1 µM (%)	MAO-A inhibition at 100 µM (%)	MAO-B inhibition at 10 µM (%)	MAO-B inhibition pIC_50_	CYP1A2 inhibition ∼ pIC_50_	ER binding pIC_50_	Aromatase inhibition ∼ pIC_50_
**1**	16	68	88	6.3 ± 0.23	27	4	48	N/A	4.07	N/B	N/I
**2**	26	69	83	6.2 ± 0.11	7	−6	16	N/A	4.48	N/B	N/I
**3**	7	47	76	5.9 ± 0.04	42	4	50	N/A	5.15	N/B	N/I
**4**	47	84	95	6.8 ± 0.05	16	N/A	64	N/A	6.89	N/B	N/I
**5**	6	18	9	N/I	37	−1	53	N/A	4.66	5.50 ± 0.13	N/I
**6**	14	23	27	5.4 ± 0.14	31	−1	101	6.39 ± 0.06	4.52	6.15 ± 0.11	N/I
**7**	16	39	62	5.8 ± 0.12	13	−11	95	6.07 ± 0.04	5.82	N/B	N/I
**8**	1	1	N/A	N/I	N/A	13	26	N/A	4.89	6.10 ± 0.07	N/I
**9**	0	3	N/A	N/I	N/A	25	86	6.00 ± 0.03	3.77	5.90 ± 0.10	N/I
**10**	1	1	N/A	N/I	N/A	N/A	11	N/A	6.94	N/A	7.11

N/I: no inhibition; N/B: no binding; N/A: not available.

^a^Average of two measurements, except for compounds **8** and **9**.

^b^Average of three measurements, except for compounds **8** and **9**. Controls: HSD1 = compound No. 21 (73.1–79.7% 10 nM or 91.3–95.3% 100 nM;^10^); HSD2 = in-house reference compound HM2001 = 3–(4-Chlorophenoxy)-5,7-dihydroxychromen-4-one (62.3% 100 nM or 100.0% 1 µM); MAO-A = clorgyline (101%); MAO-B = pargyline (103%; pIC_50_ = 7.22 ± 0.07); CYP1A2 = fluvoxamine (IC_50_ = 0.1 µM); ER: oestradiol (IC_50_ = 5.7 nM (kit reference)/0.26 ± 0.09 nM or pIC_50_ = 9.58 ± 0.12 (measured); aromatase = finrozole (IC_50_ = 0.2–0.5 µM).

Altogether, nine 3-phenylcoumarin analogues with varying coumarin and 3-phenyl ring substituents (R1–R6 positions; Figure 2) were synthesised (Table 1). Five of the analogues produced ≥62% HSD1 inhibition at 5 µM and, furthermore, three of them elicited ≥68% inhibition even at 1 µM (estimated pIC_50_ ≥ 6.2). The docking-based structure-activity relationship (SAR) analysis indicates that the potent analogues mimic steroid binding (Figure 1(D)). A cross-reactivity profile, covering HSD2, monoamine oxidases A (MAO-A) and B (MAO-B), ER, cytochrome P450 1A2 (CYP1A2), and aromatase (or CYP19A1), were built for each analogue. Importantly, the substitution of the 3-phenyl ring with the 3-imidazole ring in the coumarin core, assures strong and selective aromatase inhibition.

In short, the coumarin-based compounds have potential for lowering E_2_ levels needed in battle against diseases, such as breast cancer or endometriosis by blocking either the aromatase pathway or the sulphatase pathway.

## Methods

### Chemical procedure

All reactions were carried out using commercial materials and reagents without further purification unless otherwise noted. Reaction mixtures were heated by the CEM Discovery microwave apparatus. All reactions were monitored by thin layer chromatography (TLC) on silica gel plates. ^1^H NMR and ^13^C NMR data were recorded on a Bruker Avance 400 MHz spectrometer or Bruker Avance III 300 MHz spectrometer (Bruker, Billerica, MA). Chemical shifts are expressed in parts per million values (ppm) and are designated as singlet (s), broad singlet (br s), doublet (d), double doublet (dd), double double doublet (ddd), and triplet (t). Coupling constants (*J*) are expressed as values in hertz (Hz). The HRMS mass spectra were recorded using Micromass LCT ESI-TOF equipment (Waters Corporation, Milford, MA). Elemental analyses were done with Elementar Vario EL III elemental analyser (Elementar-Straße 1, Langenselbold, Germany). The 3-phenylcoumarin analogues were synthesised using Perkin–Oglialor condensation reaction. The method was developed from the earlier published procedures and transferred to microwave reactor.

**Scheme 1.**
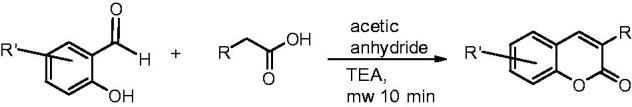
The synthesis of 3-phenylcoumarin analogues and 3-imidazolecoumarin.

Experimental data for 7-hydroxy-3–(4-fluorophenyl)-2H-chromen-2-one (**5**; Figure 2), 7-hydroxy-3–(4-methoxyphenyl)-2H-chromen-2-one (**8**; Figure 2) and 7-hydroxy-3–(4-hydroxyphenyl)-2H-chromen-2-one (**9**; Figure 2) have been published^14^. However, the synthesis steps are detailed below for other derivatives studied here (**1**, **2**, **3**, **4**, **6**, **7** and **10**Figure 2; Scheme 1). Of these **1–4** have also been synthesised earlier by others prior to this study^15–18^. 2; Scheme 1).

A typical procedure (Scheme 1): A mixture of salicylaldehyde derivative (2 mmol) and phenylacetic acid derivative (2.1 mmol), acetic acid anhydride (0.6 ml), and triethylamine (0.36 ml) were placed in a microwave reactor tube and this mixture was heated at 100–170 °C with microwave apparatus for 10–20 min. After cooling, 2 ml of 10% NaHCO_3_ solution was added and the precipitate was filtered, dried, and recrystallised from ETOH/H_2_O or acetone/H_2_O mixture. The acetyl group(s) were removed by treating the compound with MeOH/NaOH(aq) solution for 30–60 min at r.t. The solution was acidified with HCl(aq,) and the precipitate was filtered and recrystallised if needed.

Based on the elemental analysis and/or ^1^H-NMR the purity of compounds was >95%.

**8-hydroxy-3–(4-methoxyphenyl)-2*H*-chromen-2-one (1)**^15^. In the first step 8-acetoxy-3–(4-methoxyphenyl)-2*H*-chromen-2-one was obtained. Yield 85%; ^1^H-NMR (400 MHz, d^6^-DMSO) *δ*: 2.40 (s, 3H, CH_3_C(O)O-Ph), 3.80 (s, 3H (CH_3_O-Ph), 7.02 (d, 2H, *J*^3^ = 8.1 Hz, H-3′, H-5′), 7.37 (t, 1H, *J*^3^ = 7.6 Hz, H-6), 7.43 (d, *J*^3^ = 7.7 Hz, 1H, H-7), 7.64–7.69 (m, 3H, H-5, H-2′, H-6′), 8.11 (s, 1H, H-4); ^13 ^C-NMR (100.6 MHz, d^6^-DMSO) *δ*: 20.33, 55.20, 113.70, 120.85, 124.41, 124.65, 125.87, 126.50, 126.80, 129.82, 136.70, 138.88, 144.38, 158.83, 159.73 and 168.38. HRMS(ESI): calcd for C_18_H_14_O_5_Na_1_ [M + Na]^+^: 333.07389, found 333.07580. Elemental anal. for C_18_H_14_O_5,_ calc. C% 69.67, H% 4.55, found C% 69.53, H% 4.55. In the second step, 8-hydroxy-3–(4-methoxyphenyl)-2*H*-chromen-2one was obtained. Yield 81%; ^1^H-NMR (400 MHz, d^6^-DMSO) *δ*: 3.80 (s, 6H (CH_3_O−), 7.01 (d, *J*^3^ = 8.9 Hz, 2H, H-3′,H-5′), 7.08 (dd, 1H, *J*^3^ = 7.0 Hz, *J*^4^ = 2.6 Hz, H-7), 7.12–7.18 (m, 2H, H-5, H-6), 7.70 (d, 2H *J*^3^ = 8.9 Hz, H-2′,H-6′), 8.11 (s, 1H, H-4), 10.19 (s, 1H, Ph-OH). ^13 ^C-NMR (100.6 MHz, d^6^-DMSO) *δ*: 55.21, 113.64, 117.64 118.39, 120.55, 124.45, 126.22, 126.91, 129.79, 139.52, 141.42, 144.26, 159.54 and159.76. HRMS(ESI)): calcd for C_16_H_12_O_4_Na_1_ [M + Na]^+^: 291.06333, found 291.06180. Elemental anal. for C_16_H_12_O_4,_ calc. C% 71.26, H% 4.51, found C% 71.64, H% 4.51.

**6-hydroxy-3–(4-hydroxyphenyl)-2*H*-chromen-2-one (2)**^19^. In the first step 4–(6-acetoxy-2-oxo-2*H*-chromen-3-yl)phenyl acetate was obtained. Yield 90%; ^1^H-NMR (300 MHz, d^6^-DMSO) *δ*: 2.30 (s, 3H, CH_3_CO(O)-Ph), 2.31 (s, 3H, CH_3_CO(O)-Ph), 7.23 (d, 2H, *J*^3^ = 8.8 Hz, H-2′, H-6′), 7.40 (dd, *J*^3^ = 8.9 Hz, *J*^4^ = 2.7 Hz, 1H, H-7), 7.49 (d, 1H, *J*^3^ = 8.9 Hz, H-8), 7.55 (d, 1H, *J*^4^ = 2.6 Hz, H-5), 7.76 (d, 2H, *J*^3^ = 8.8 Hz, H-3′, H-5′), 8.24 (s, 1H, H-4); ^13 ^C-NMR (75.5 MHz, d^6^-DMSO) *δ*: 20.73, 20.82, 116.97, 119.90, 120.77, 121.67, 125.48, 126.67, 129.74, 131.95, 139.84, 146.43, 150.43, 150.76, 159.51, 169.10 and 169.22. In the second step, 6-hydroxy-3–(4-hydroxyphenyl)-2*H*-chromen-2-one was obtained. Yield 85%; ^1^H-NMR (400 MHz, d^6^-DMSO) *δ*: 6.83 (d, 2H, *J*^3^ = 8.8 Hz, H-3′, H-5′), 6.99 (dd, 1H, *J*^3^ = 8.8 Hz, *J*^4^ = 2.9 Hz, H-7), 7.06 (d, 1H, *J*^4^ = 2.8 Hz, H-5), 7.24 (d, 1H, *J*^3^ = 8.9 Hz, H-8), 7.57 (d, 2H, *J*^3^ = 8.7 Hz, H-2′, H6′), 8.04 (s, 1H, H-4); ^13 ^C-NMR (75.5 MHz, d^6^-DMSO) *δ*: 112.29, 115.00,116.59, 119.15, 120.24, 125.40, 126.71 129.86, 138.51, 146.03, 153.77, 157.90 and 160.13. HRMS(ESI)): calcd for C_16_H_11_F_1_O_4_Na_1_ [M + Na]^+^: 277.0477, found 277.0461.

**3–(3-hydroxyphenyl)-2*H*-chromen-2-one (3)**^20^. In the first step, 3–(2-oxo-2H-chromen-3-yl)phenyl acetate was obtained. Yield 87%; ^1^H-NMR (400 MHz, d^6^-DMSO) *δ*: 2.30 (s, 3H, CH_3_C(O)O-Ph), 7.20 (ddd, 1H, *J*^3^ = 9.0 Hz, *J*^4^ = 2.2 Hz, *J*^4′^ = 2.3 Hz, H-6′), 7.39 (t, 1H, *J*^3^ = 7.6 Hz, H-5′), 7.44 (d(broad), 1H, *J*^3^ = 8.3 Hz, H-4′), 7.49–7.53 (m, 2H, H-6, H-2′), 7.62–7.66 (m, 2H, H-7, H-8) 7.79 (dd, 1H, *J*^3^ = 8.7 Hz, *J*^4^ = 1.5 Hz, H-5), 8.32 (s, 1H, H-4); ^13 ^C-NMR (100 MHz, d^6^-DMSO) *δ*: 20.86, 115.90, 119.38, 121.81, 122.17, 124.68, 125.69, 125.90, 128.81, 129.31, 131.98, 135.99, 141.13, 150.30, 153.02, 159.51 and 169.23. In the second step, 3–(3-hydroxyphenyl)-2*H*-chromen-2-one was obtained. Yield 74%; ^1^H-NMR (300 MHz, d^6^-DMSO) *δ*: 6.83 (ddd, 1H, *J*^3^ = 8.1 Hz, *J*^4^ = 2.2 Hz, *J*^4′^ = 2.4 Hz, H-4′), 7.11–7.18 (m, 2H, H-2′, H-6′), 7.26 (t, 1H, *J*^3^ = 7.9, H-5′), 7.37 (ddd, 1H, *J*^3^ = 7.6 Hz, *J*^4^ = 1.1 Hz, *J*^4′^= 1.1 Hz, H-6), 7.42 (d, *J*^3^ = 8.3 Hz, H-8), 7.61 (ddd, *J*^3^ = 7.3 Hz, *J*^4^ = 1.6 Hz, *J*^4^′ = 2.6 Hz H-7), 7.83 (dd, 1H, *J*^3^ = 8.7 Hz, *J*^4^ = 1.5 Hz, H-5), 8.20 (s, 1H, H-4), 9.54 (s, 1H,Ph-OH); ^13 ^C-NMR (75.5 MHz, d^6^-DMSO) *δ*: 115.45, 115.59, 115.76, 119.13, 119.43, 124.50, 126.86, 128.60, 129.20, 131.60, 135.79, 140.32, 152.87, 157.06, and 159.54. HRMS (ESI): Calcd for C_15_H_10_O_4_Na_1_ [M + Na]^+^: 261.05276, found 261.04980.

**6-chloro-3–(3-hydroxyphenyl)-2*H*-chromen-2-one (4)**^21^. In the first step, 3–(6-chloro-2-oxo-2H-chromen-3-yl)phenyl acetate was obtained. Yield 85%; ^1^H-NMR (400 MHz, d^6^-DMSO) *δ*: 2.30 (s, 3H, CH_3_C(O)O-Ph), 7.22 (ddd, 1H, *J*^3^ = 8.0 Hz, *J*^4^ = 2.2 Hz, *J*^4′^ = 2.3 Hz, H-6′), 7.48–7.52 (m, 3H, H-8, H-2′, H-5′), 7.62 (m, 1H, H-4′), 7.67 (dd, 1H, *J*^3^ = 8.9 Hz, *J*^4^ = 2.6 Hz, H-7), 7.88 (d, 1H, *J*^4^ = 2.6 Hz, H-5), 8.27 (s, 1H, H-4).); ^13 ^C-NMR (100 MHz, d^6^-DMSO) *δ*: 20.85, 117.96, 120.78, 121.85, 122.47, 125.93, 126.88, 127.70, 128.31, 129.41, 131.45, 135.67, 139.82, 150.30, 151.66, 159.10 and169.22. In the second step, 6-chloro-3–(3-hydroxyphenyl)-2*H*-chromen-2-one was obtained. Yield 80%; %; ^1^H-NMR (400 MHz, d^6^-DMSO) *δ*:), 6.84 (ddd, 1H, *J*^3^ = 8.0 Hz, *J*^4^ = 2.4 Hz, *J*^4′^ = 2.3 Hz, H-6′), 7.10–7.15 (m, 2H), 7.27 (t, 1H, *J*^3^ = 7.9 Hz, H-5′), 7.47 (d, 1H, *J*^3^ = 8.9 Hz, H-8), 7.65 (dd, 1H, *J*^3^ = 8.3 Hz, *J*^4^ = 2.6 Hz, H-7), 7.90 (d, 1H, *J*^4^ = 2.5 Hz, H-5), 8.17 (s, 1H, H-4), 9.57 (s, 1H, Ph-OH); ^13 ^C-NMR (75.5 MHz, d^6^-DMSO) *δ*: 115.43, 115.85, 117.79, 119.13, 120.84, 127.53, 127.99, 128.18, 129.26, 131.09, 135.44, 139.03, 151.50, 157.08 and 159.11. HRMS (ESI): Calcd for C_15_H_9_Cl_1_O_3_Na_1_ [M + Na]^+^: 295.01379, found 295.01380.

**3–(3-fluoro-4-hydroxyphenyl)-7-methoxy-2*H*-chromen-2-one (6).** In the first step, 2-fluoro-4–(7-methoxy-2-oxo-2H-chromen-3-yl)phenyl acetate was obtained. Yield 75%; ^1^H-NMR (400 MHz, d^6^-DMSO) *δ*: 2.35 (s, 3H, CH_3_C(O)O-Ph), 3.88 (s, 3H, CH_3_O-Ph), 6.99 (dd, 1H, *J*^3^ = 8.6 Hz, *J*^4^ = 2.4 Hz, H-6), 7.05 (d, 1H, *J*^4^ = 2.4 Hz, H-8), 7.37 (t, 1H, *J* = 8.3 Hz, H-6′), 7.62 (d, *J* = 8.5 Hz, 1H, H-5′), 7.68 (d, *J* = 8.6 Hz, 1H, H-5), 7.74 (dd, *J*^H-F^ = 12.1 Hz, *J*^4^= 2.0 Hz, H-3′), 8.31 (s, 1H, H-4); ^13 ^C-NMR (100 MHz, d^6^-DMSO) *δ*: 20.19, 55.97, 100.25, 112.79, 116.35 (d, *J*^C–F^ = 20 Hz), 121.02 (d, *J*^C-F^ = 1.9 Hz), 123.83, 124.79 (d, *J*^C-F^ = 3.2 Hz), 129.86, 134.24 (d, *J*^C–F^ = 7.7 Hz), 137.20 (d, *J*^C-F^ = 13.1 Hz), 141.55, 153.00 (*J*^C-F^ = 246.1 Hz), 154.92, 159.65 and 162.69, 168.19. In the second step, 3–(3-fluoro-4-hydroxyphenyl)-7-methoxy-2*H*-chromen-2-one was obtained. Yield 70%; ^1^H-NMR (400 MHz, d^6^-DMSO) *δ*: 3.87 (s, 3H, CH_3_O-Ph), 6.96–7.03 (m, 3H, H-6, H-8, H-5′), 7.41 (d, 1H, *J*^3^ = 8.4, H-6′), 7.57 (dd, 1H, *J*^H-F^ = 13.1 Hz, *J*^4^ = 2.2 Hz (H-H), 1H, H-2′), 7.66 (d, 1H, *J*^3^ = 8.4, H-5), 8.18 (s, 1H, H-4), 10.09 (s, 1H, Ph-OH). ^13 ^C-NMR (75.5 MHz, d^6^-DMSO) *δ*: 55.91, 100.16, 112.61, 113.04, 115.95 (d, *J*^C-F^ = 20 Hz), 117.37 (d, *J*^C-F^ = 3.3 Hz), 121.78 (*J*^C-F^ = 2.0 Hz), 124.54 (d, *J*^C-F^ = 3.0 Hz), 126.08 (d, *J*^C-F^ = 7.0 Hz), 129.49, 139.62, 145.0 (*J*^C-F^ = 13 Hz), 150.46 (d, *J*^C-F^ = 240 Hz), 154.52, 159.87 and 162.19. HRMS (ESI): Calcd for C_16_H_11_F_1_O_4_Na_1_ [M + Na]^+^: 309.0539, found 309.0553.

**3–(3-fluoro-4-hydroxyphenyl)-6-methoxy-2*H*-chromen-2-one (7).** In the first step, 2-fluoro-4–(6-methoxy-2-oxo-2H-chromen-3-yl)phenyl acetate was obtained. Yield 66%; ^1^H-NMR (400 MHz, d^6^-DMSO) *δ*: 2.33 (s, 3H, CH_3_C(O)O-Ph), 3.82 (s, 3H (CH_3_O-Ph), 7.23 (dd, 1H, *J*^3^ = 9.0 Hz, *J*^4^ = 3.0 Hz, H-7), 7.30 (d, 1H, *J*^4^ = 3.0 Hz, H-5), 7.35 (d, 1H, *J*^3^ = 9.2 Hz, H-8), 7.61 (d, 1H, *J*^3^ = 8.5 Hz, H-5′), 7.75 (dd, 1H, *J*^H-F^ = 12.0 Hz, *J*^4^ = 1.7 Hz (H-H), 1H, H-2′), 8.30 (s, 1H, H-4); ^13 ^C-NMR (100.6 MHz, d^6^-DMSO) *δ*: 20.22, 55.69, 110.83, 116.67, 117.02, 119.66, 123.96, 125.10, 135.96, 141.18, 147.44, 151.78, 154.23, 155.70, 159.53 and 168.21. In the second step, 3–(3-fluoro-4-hydroxyphenyl)-6-methoxy-2*H*-chromen-2-one was obtained. Yield 71%; ^1^H-NMR (400 MHz, d^6^-DMSO) *δ*: 3.81 (s, 3H (CH_3_O-Ph), 7.02 (dd, 1H, *J*^3^ = 9.2 Hz, H-6′), 7.18–7.28 (m, 1H, H-5, H-7), 7.35 (d, *J*^3^ = 9.0 Hz, H-8), 7.42 (d, 1H, *J*^3^ = 8.4 Hz, H-5′), 7.57 (dd, 1H, *J*^H-F^ = 13.0 Hz, *J*^4^ = 2.2 Hz (H-H), 1H, H-2′), 8.17 (s, 1H, H-4), 10.19 (s, 1H, Ph-OH); ^13 ^C-NMR (100.6 MHz, d^6^-DMSO) *δ*: 55.66, 110.59, 116.67, 117.02, 119.66, 123.96, 125.10, 135.96, 141.18, 147.44, 151.78, 154.23, 155.70, 159.53 and 168.21. HRMS (ESI): Calcd for C_16_H_11_F_1_O_4_Na_1_ [M + Na]^+^: 309.0539, found 309.0521.

**3–(1H-imidazol-1-yl)-2H-chromen-2-one (10).** Yield: 39% light brown solid; *R*_f _=0.18 (EtOAc); ^1^H-NMR (300 MHz, d^6^-DMSO) *δ*: 7.10 (br s, 1H, H-4′), 7.44 (apparent td, *J*^3 ^= 7.5 Hz, *J*^4 ^=1.0 Hz, 1H, H-6), 7.51 (d, *J*^3^=8.3 Hz, 1H, H-8), 7.64–7.70 (m, two overlapping signals, 2H, H-7 and H-5′), 7.77 (dd, *J*^3^=7.7 Hz, *J*^4 ^=1.5 Hz, 1H, H-5), 8.16 (br s, 1H, H-2′), 8.34 (s, 1H, H-4). ^13 ^C-NMR (75 MHz, d^6^-DMSO) *δ*: 116.06 (***C***-H8), 118.51 (H5-C-***C***-C-H4), 119.57 (***C***-H5′), 123.37 (N-***C***-C = O), 125.09 (***C***-H6), 128.63 (***C***-H4′), 128.80 (***C***-H5), 131.87 (***C***-H7), 132.97 (***C***-H4), 137.12 (N-***C***(-H2′)=N), 151.78 (H8-C-***C***-O), 156.83 (***C***=O). IR (KBr): 1727, 1708, 1630, 1608, 1486, 1318, 1083 and 760. ESI-MS: *m/z* (rel. abund. %): calculated for (M + Na^+^) = 235.0478, measured 235.0476, *Δ* = 0.2 mDa. Elemental analysis for C_12_H_8_N_2_O_2_: calc. C% 67.92, H% 3.80, N% 13.20, found C% 67.49, H% 3.72 and N% 13.13. Mp. 180–182 °C.

### 17-β-Hydroxysteroid dehydrogenase 1 and 2

The inhibition was determined by HPLC using recombinant human HSD1 and HSD2 proteins as described in a prior study^10^. In short, recombinant human HSD1 and HSD2 were produced in Sf9-insect cells. The assay was performed in a final volume of 0.2 ml buffer (20 mM KH_2_PO_4_, 1 mM EDTA, pH 7.4) containing 0.1 mg/ml protein, 1 mM cofactor (NADPH for HSD1, NAD for HSD2), 30 nM substrate oestrone or oestradiol, 800,000 cpm/ml of tritium labelled oestrone ([3H]-E1) or oestradiol ([3H]-E2), and inhibitors concentrations in the range of 0.1–5.0 mM. Triplicate samples were incubated for 25 min at the room temperature. After incubation, the reaction was stopped by addition of 20 ml 10% trichloroacetic acid per sample. After incubation the substrate and the product of enzymatic conversion [3H]-E1 and [3H]-E2 were separated and quantified by HPLC (Alliance 2790, Waters, Milford, MA) connected to an online counter (Packard Flow Scintillation Analyser; Perkin Elmer Inc., Waltham, CA). The ratio of [3H]-E1 converted to [3H]-E2 or vice versa determines the conversion percentage of the samples. Inhibition was measured in three concentrations (100 nM, 1, and 5 µM) in order to follow the progression of inhibition efficiencies. Inhibition efficiencies of the tested inhibitors were calculated by comparing the conversion percentages of the samples including inhibitors with those of conversion controls (without inhibitors). The pIC_50_ average values and their standard errors were estimated from three measurements at 1 µM.

### Aromatase

Aromatase (CYP19A1) activity was measured as described previously^22^ by using human placental microsomes and 50 nM [3H]-androstenedione as a substrate and inhibitor concentrations in the range of 60–1000 nM. Aromatase activities were measured as released [3H]-H_2_O in Optiphase Hisafe 2 scintillation liquid (Perkin Elmer, Waltham, MA) with a Wallac 1450 MicroBeta Trilux scintillation counter (Perkin Elmer, Waltham, MA). As a positive control for aromatase inhibition, 1 µM finrozole (generous gift from Olavi Pelkonen, University of Oulu, Finland) was used.

### Monoamine oxidase A and B

The protein in addition to the reagents for the chromogenic solution (vanillic acid (4-hydroxy-3-methoxylbenzoic acid, 97% purity), 4-aminoantipyrine (reagent grade), horseradish peroxidase, and the substrate tyramine hydrochloride (minimum 99% purity)) as well as the potassium phosphate buffering agents (potassium phosphate dibasic trihydrate (≥99% ReagentPlusTM) and potassium phosphate monobasic (minimum 98% purity, molecular biology tested)) were all purchased from Sigma-Aldrich (St. Louis, MO). The protocol of continuous spectrophotometric assay by Holt et al. was first used to determine the activity of the proteins^23^. The assay was performed in 0.2 M potassium phosphate buffer pH 7.6 on 94-well plates (NuncTM 96 F microwell plate without a lid, Nunc A/S, Roskilde, DK) with chromogenic solution containing 250 µM vanillic acid, 125 µM 4-aminoantipyrine and 2 U/ml horseradish peroxide in the total assay volume of 200 µl. The protein was first incubated for 30 min at 37 °C in the chromogenic solution and then the substrate tyramine was introduced at 0.5 mM final plate concentration completing the assay volume. The activity measurement using multilabel reader (VictorTM X4, 2030 Multilabel Reader, PerkinElmer, Waltham, MA) at A_490_ immediately followed and the plates were read 300 times every 15 s using 1 s exposure time. The assay should produce absorbance change of ∼0.35^23^. The more active MAO-A produced over 0.5 change in absorbance reaching the assay maximum in 30 min with 25 µg of protein (enzymatic activity 5.25 units) per well while MAO-B produced the expected 0.35 change in absorbance with 50 µg of protein (enzymatic activity 3.2 units) per well and reached the assay maximum in 2 h. These protein concentrations were selected to be used to analyse the molecules **1**–**9**. The analysis conditions followed the above-described assay protocol^23^ and the activity of tested molecules was measured at 100 µM for MAO-A and at 10 µM for MAO-B. The analysis was performed as single point measurements and the signal was read by the same instrument at the expected assay maximum indicated by the activity measurements, at 30 min for MAO-A and at 2 h for MAO-B, respectively. Clorgyline was used as MAO-A and pargyline as MAO-B inhibitor control. Both of the control inhibitors provided 100% inhibition at the assay concentration of the test molecules. In addition, pIC_50_ values were determined for MAO-B inhibition using duplicated dilution series and the pIC_50_ value calculated for MAO-B inhibition by pargyline was 6.21. The observed activity was calculated as inhibition percentage (Table 1). The pIC_50_ values were calculated with GraphPad Prism version 5.03 (GraphPad Software Inc., San Diego, CA).

### Oestrogen receptor

The pIC_50_ values of the molecules (Table 1) were measured using green PolarScreen^™^ ER Alpha Competitor Assay (Life Technologies, Carlsbad, CA) kit, following the protocol provided by the manufacturer as previously described^14^. The final concentration of the molecules ranged between 0.0007 and 10,000 nM in the prepared dilution series. The molecules were combined with 25 nM ERα and 4.5 nM fluormone in the assay buffer and placed on black low volume 384-well assay plate with NBS surface (Corning Inc., Corning, NY). After mixing the assay plate, it was incubated for 2 h at the room temperature. The fluorescence polarisation was then measured using excitation wave length 485 and emission wave length 535 with bandwidths of 25/20 nm on a 2104 EnVision^®^ Multilabel Plate Reader which had EnVision Workstation version 1.7 (PerkinElmer, Waltham, MA).

### Cytochrome P450 1A2

Inhibition of CYP1A2 activity was determined using commercial heterologously expressed human CYP1A2 enzyme (Corning Inc., Corning, NY) essentially as described previously^24^.

### Molecular docking

**Figure 3.**
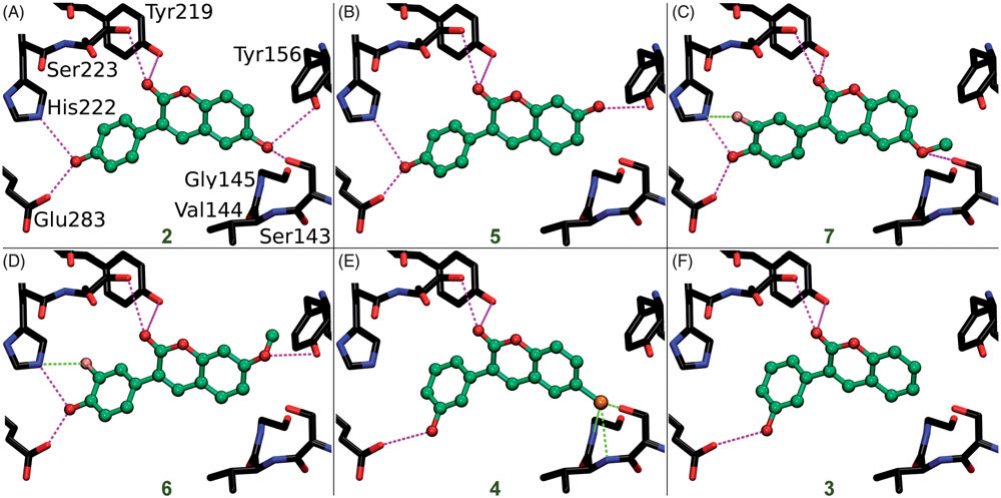
The canonical binding modes of 3-phenylcoumarin analogues inhibiting 17-β-hydroxysteroid dehydrogenase 1. The H-bonding (magenta dotted lines) and halogen bonding/favourable electrostatic (green dotted lines) interactions of (A) compounds **2**, (B) **5**, (C) **7**, (D) **6**, (E) **4**, and (F) **3** shown as suggested by docking. The active site residues of HSD1 enzyme (stick models with black backbone) bonding with the 3-phenylcoumarin analogues (ball-and-stick models with green backbone) are shown. The fluorine atom in **6** (D) and **7** (C) as well as the chlorine atom in **4** (E) are shown with pink and orange colour, respectively. Note that the His222 side chain is set epsilon protonated in order to facilitate H-bonding with the analogues. This is the opposite arrangement, if compared to the delta protonation of His222 suggested by the original E2B-bound HSD1 X-ray crystal structure (PDB: 3HB5)^25^.

**Figure 4.**
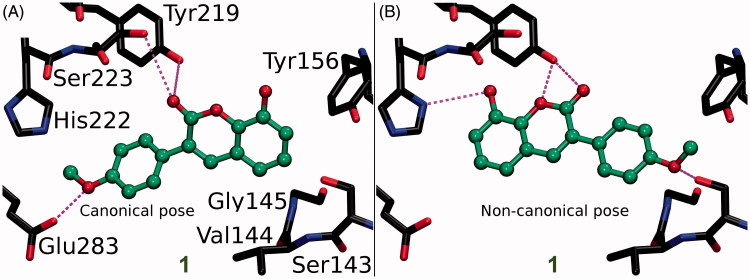
The canonical vs. non-canonical binding mode of compound **1**. (A) The “canonical” binding mode at the HSD1 active site, likely adopted by the other 3-phenylcoumarin analogues (Figure 3), is not suggested for compound **1** based on the docking-based SAR analysis; (B) instead, an alternative “non-canonical” pose is proposed for this potent inhibitor. Note that the His222 side chain is set delta protonated to facilitate H-bonding with the analogue’s R3-hydroxyl. See Figure 3 for further details.

The small-molecule ligands (Figure 2), including their probable tautomeric states and 3D conformers, were built using LIGPREP, CONFGEN, and MACROMODEL modules in MAESTRO 2016–3 (Schrödinger, LLC, New York, NY, 2016) to match pH 7.4. The compounds were docked to the X-ray crystal structures of HSD1 (PDB: 3HB5^25^; Figures 3 and 4), aromatase (PDB: 3EQM^26^; Figure 6(C)), MAO-B (PDB: 2V61^27^; Figure 6(A)) and CYP1A2 (PDB: 2HI4^28^; Figure 6(B,C)) with the PANTHER protocol^29^, where the ligand-binding site is described as a negative image, and the shape and electrostatic potentials of the Panther-models and ligand conformations are compared using SHAEP^30^.

### Figure preparation

**Figure 5.**
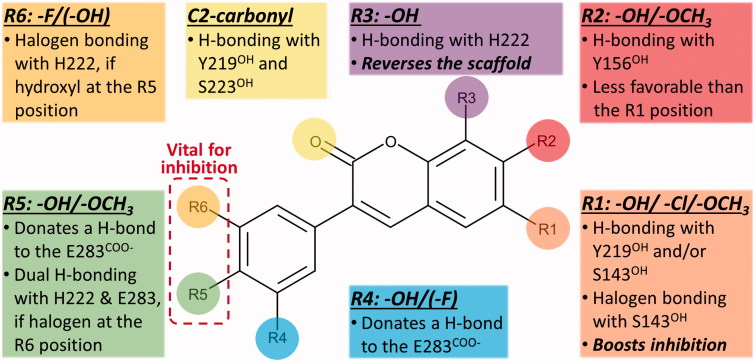
The docking-based structure-activity relationship analysis of the 3-phenylcoumarin analogues with 17-β-hydroxysteroid dehydrogenase 1.

**Figure 6.**
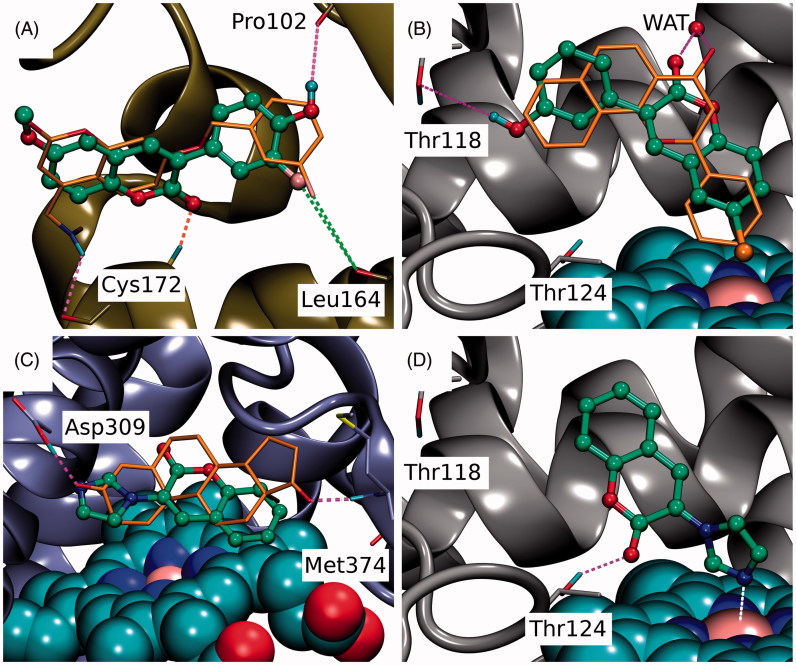
The binding of coumarin derivatives with aromatase, monoamine oxidase B and CYP1A2. (A) With the MAO-B (yellow cartoon), the docked pose of **6** demonstrates the analogous hydrophobic packing characteristic of the 3-phenylcoumarin analogues with the inhibitor C18 (stick model with orange backbone; PDB: 2V61)^27^. Notably, the R6-positioned polar group, fluorine in particular, improves the inhibition by forming a halogen bond with the Leu164°. (B) The docked pose of **4** (ball-and-stick model with green backbone) at the active site of CYP1A2 (grey cartoon) mimics α-naphthoflavone (stick model with orange backbone; PDB: 2HI4)^28^. Additionally, the R1-chlorine packs against the haeme and the C2-carbonyl and R4-hydroxyl, respectively, H-bond with crystal water (wat) and the Thr118^OH^. (C) Based on docking, **10** (ball-and-stick model with green backbone) aligns similarly on top of the haeme (CPK model with cyan carbon atoms) in the active site of aromatase (magenta cartoon) as the androstenedione (stick model with orange backbone). Unlike the 3-phenylcoumarins the compound **10** has an acceptor group or the N3’ in the imidazole ring capable of H-bonding with the neutral Asp309 (PDB: 3EQM)^26^ and, thus, 3-imidazolecoumarin is a potent aromatase inhibitor. Alternatively, the N3’ of **10** could be coordinated with the haeme (not shown). (D) The coumarin ring of **10** is aligned in a way that its C2-carbonyl accepts an H-bond from the Thr124^OH^. Moreover, the deprotonated and electronegative N3’ of 3-imidazole ring is likely coordinated with the positively charged iron in the haeme (CPK model with cyan carbon atoms). See Figure 3 for further details.

Figures 2 and 5 showing 2D structures of the 3-phenylcoumarin scaffold and the analogues are drawn with BIOVIA Draw 2016 (Dassault Systèmes, San Diego, CA, 2016). Figures 1, 3, 4 and 6 are prepared using BODIL^31^ and VMD 1.9.2^32^.

## Results and discussion

### Computer-aided drug discovery

Whether the small-molecule design originates from automated virtual screening schemes, expert *de novo* work^33^ or combination of the two, the computer-aided drug discovery (CADD) requires experimental verification^14^^,^^34^. This is achieved by pairing biochemical activity testing with, for example X-ray crystallographic studies^35^, site-directed mutagenesis experiments^36^^,^^37^, and/or “mutating” the lead compounds into diverse libraries of closely-related analogues using organic synthesis^38–40^. The latter approach was applied here to demonstrate that 3-phenylcoumarin (Figure 2) is a suitable non-steroidal scaffold for building potent and selective HSD1 inhibitors.

### Inhibitor design hypothesis

Based on a detailed analysis of the known inhibitors, 3-phenylcoumarin was chosen as a suitable scaffold for designing non-steroidal HSD1-specific inhibitors *de novo*. The analogue ring system alignment at the active site of HSD1 would mimic the hydrophobic packing of the steroid ring (e.g. inhibitor E2B; PDB: 3HB5 (25); Figure 1(D)). The coumarin ring would align in an orientation that allows its C2-carbonyl to form direct hydrogen bonds (or H-bonds) with the hydroxyl groups of Tyr219 (or Tyr219^OH^) and/or Ser223 (or Ser223^OH^; Figures 3 and 4). The coumarin ring could flip also sideways, if Arg258 side chain would rotate into the active site to interact with the C2-carbonyl. The probability of this rotamer adjustment is difficult to estimate due to missing density data on the relevant X-ray crystal structure (PDB: 1EQU)^41^. Beyond this hypothesis, the plan was to establish and improve the 3-phenylcoumarin binding and HSD1 inhibition by introducing a number of polar (hydroxyl/methoxy/halogen) moieties for the 3-phenyl ring’s R4–R6 and the coumarin ring’s R1–R3 positions (Figure 2; Table 1).

### Inhibition of 17-β-hydroxysteroid dehydrogenase 1 by the 3-phenylcoumarin analogues

The activity measurements (Table 1) indicated that the 3-phenylcoumarin is indeed a suitable scaffold for building HSD1 inhibitors. The dissimilarities in the inhibition levels between the analogues arise from their R1–R6 substituents (Figure 1). Five analogues produced ≥62% HSD1 inhibition at 5 µM (Table 1). Moreover, analogues **1**, **2** and **4** produced ≥68% inhibition (estimated pIC_50_ ≥ 6.2) at 1 µM. The most potent inhibitor **4** produced 47% inhibition even at 100 nM. Rest of the analogues elicited much weaker inhibition at 100 nM. The inhibition was consistently, regardless of the concentration, more modest for analogues **3**, **5**, **6** and **7** than for the three most potent analogues. In contrast, analogues **8** and **9** did not block the HSD1 (Table 1).

### Scaffold hopping: 3-phenylcoumarin vs. steroid alignment

Due to the plasticity of the catalytic site, full understanding of the structural basis of the HSD1 inhibition or the selectivity is challenging. The ring systems of the 3-phenylcoumarin could mimic the steroid ring positioning in four different ways, if only the hydrophobic packing is considered. To address this issue, a specifically tailored docking protocol was utilised^29^^,^^42^^,^^43^ for predicting how the analogues bind and elicit the inhibition (Figure 3). This docking-based SAR analysis point out how the R1–R6 moieties (Figure 2) affect the HSD1 binding (Table 1) and inhibition (Figures 3 and 4).

Coumarin (2H-chromen-2-one) contains a bicyclic structure of phenyl ring fused to a six-member ring with 1- and 2-positioned oxygen atom and carbonyl group, respectively (Figure 2). The 3-phenyl is tilted in relation to the coumarin ring as indicated by the small-molecule X-ray crystallography (CSD: QECNUJ)^44^. The binding of the 3-phenylcoumarin analogues is predicted to mimic closely the pose and hydrophobic packing of the E2B’s steroid ring at the active site of HSD1 (Figure 1(D)). The ring positioning is likely highly similar or “canonical” for the HSD1 analogues (Figure 3), except for **1** (Figure 4). Moreover, both the Ser223^OH^ and the Tyr219^OH^ are predicted to H-bond with the coumarin’s C2-carbonyl (Figures 3 and 4).

### R1 position is important for strong 3-phenylcoumarin inhibition

A docking-based SAR analysis (Figure 5) explains the atomistic determinants of the HSD1 inhibition for each analogue.

The strong potency of **2** (Figure 2; Table 1) reflects its ability to form well-coordinated H-bonds between the proximal R1/R5-hydroxyl groups and the residues lining both ends of the binding site (Figure 3(A)). The R1-hydroxyl of the coumarin ring H-bonds with the main chain oxygen of Tyr156 (or Tyr156^O^) and the Ser143 side chain. Furthermore, the main chain nitrogen of Val144 (or Val144^N^), Gly145^N^, and Cys186^O^ are favourably positioned in relation to the analogue’s R1-hydroxyl group. In turn, the R5-hydroxyl H-bonds with the carboxyl group of Glu283 (or Glu283^COO-^) and, reciprocally, accept an H-bond from the epsilon position of His222.

When the R1-hydroxyl of **2** is switched to the R2 position at the coumarin ring in **5** (Figure 2), the HSD1 inhibition lowers dramatically (Table 1). This highlights the importance of the R1 position for the 3-phenylcoumarin binding as the overall alignment of **2** and **5** is likely similar despite the switch (Figure 3(A,B)). Although the R2-hydroxyl is able to H-bond with the Tyr156^OH^, it is evident that the R1-hydroxyl of **2** form stronger interactions with the close-by residues than the R2-hydroxyl (Figure 3(A,B)). The R5-hydroxyl of **5** assumes the same dual H-bonding role with Glu283 and His222 as the equivalent hydroxyl of **2**; assuring inhibition despite the R1/R2-hydroxyl switch (Table 1).

Replacing the R1-hydroxyl with a methoxy lowers the HSD1 inhibition considerably (Figure 5). This effect is apparent when **7** (Figure 2) is compared to **2** (Table 1). Although the R1-methoxy is H-bonding with the Ser143^OH^ in the docked pose (Figure 3(C)), it cannot coordinate as many or as strong interactions in this position as a hydroxyl (Figure 3(A,C)). However, the addition of R6-fluorine next to the hydroxyl offsets in part the negative effect of the R1 substitution. The fluorine is able to form a halogen bond with His222 (Figure 3(C)). In addition, the R5-hydroxyl of **7** function in the same dual H-bonding role with the side chains of Glu283 and His222 (compare to **2** and **5**; Figure 3(A–C)).

When the R1-methoxy of **7** is shifted to the R2 position in **6** (Figure 2), the inhibition is moderately reduced (Table 1). This effect is analogous to the weakening of inhibition seen in response to the R1/R2-hydroxyl switch between **2** and **5** (Figure 1(A,B)). The R5-hydroxyl of **6** H-bonds with both His222 and Glu283 and the adjacent R6-fluorine halogen bonds with His222 (Figure 3(D)). Despite the proximity of the R2-methoxy to several H-bond donors, such as the Tyr156^OH^ and the Ser143^OH^, the group cannot form as coordinated polar interactions as the R1-methoxy of **7** (Figure 3(C,D)).

The importance of the R1 position is highlighted with **4** (Figure 2) – the most potent HSD1 inhibitor of the analogues set (Table 1). Although the R4-hydroxyl of **4** donates an H-bond only to the Glu283^COO-^ (Figure 3(E)), the inhibition is strong (Table 1). This is likely due to the hydroxyl/chlorine substitution at the R1 position (Figure 3(A,E); Table 1) allowing the R1-chlorine to halogen bond with the Ser143^OH^ and potentially with the Tyr156^OH^ (Figure 3(E)). Besides, the protons of the Val144^N^ and the Gly145^N^ cater to the halogen’s negative charge. The inability of **4** to form H-bonds with both Glu283 and His222 is, therefore, likely offset by the analogue’s ability to halogen bond (Table 1).

The relatively poor potency of **3** (Figure 2; Table 1) correlates with its limited ability to H-bond (Figure 2). Although **3** is almost identical to the most potent inhibitor **4**, it lacks the R1-chlorine (Figure 3(E,F)). The Glu283^COO-^ and the Ser223^OH^ form H-bonds with the R6-hydroxyl and the C2-carbonyl, respectively (Figure 3(F)). The Tyr219^OH^, in turn, is potentially H-bonding with the C2-carbonyl. This underlines the importance of proximal groups capable of bonding at the coumarin’s R4-R6 positions for the HSD1 inhibition (Figure 2; Table 1).

### R5/R6-hydroxyl group is critical for the 3-phenylcoumarin inhibition

Analogues **2**–**7** (Figure 3; Table 1) collectively indicate that a halogen or hydroxyl at the R1 position (Figure 2) improves the HSD1 inhibition of the 3-phenylcoumarins (Table 1; Figure 5). The absolute position or even the presence of this group is, however, not essential for inhibition (Figure 2; Table 1). In contrast, if one considers only those six analogues (Figure 3), excluding **1** (Figure 4), that produces inhibition at 1 or 5 µM (Table 1), placing a hydroxyl group at the R4 or R5 position is a necessity (Figure 2).

In **8** (Figure 2), there is a hydroxyl group at the R2 position of the coumarin ring the same way as in **5**, but the lack of a hydroxyl group in the 3-phenyl ring renders the analogue unable to bond with Glu283 and His222. The loss of this dual contact is not fully compensated by the R5-fluorine and, as a result, the HSD1 inhibition is non-existent (Table 1). Further evidence of the importance of R4/R5-hydroxyl is provided by the inability of **9** (Figure 2) to prevent the HSD1 activation. The R5-methoxy of **9** cannot establish as strong H-bonding coordination for the 3-phenyl as a hydroxyl group in the “canonical” pose would. In this respect, **1** (Figure 2) is a noteworthy exception. Although the analogue’s 3-phenyl ring contains only R5-methoxy group and no hydroxyl moiety (Figure 2), it still induces strong inhibition (Table 1).

### R3-hydroxyl reverses the 3-phenylcoumarin binding

The binding of **1** is predicted to differ markedly (Figure 4) from other 3-phenylcoumarin analogues (Figure 3) producing HSD1 inhibition (Table 1; Figure 5). Instead of the “canonical” pose (Figure 4(A)), the coumarin and 3-phenyl ring systems of **1** are suggested to have reverse order or “non-canonical” positioning of at the site (Figure 4(B)) when compared to the other analogues (Figure 3). Even though this flip represents a profound change for the scaffold, it imposes only few drawbacks.

The ring systems of **1** pack against the same residues as they would in the “canonical” pose. Importantly, the R3-hydroxyl accepts an H-bond from the delta position of His222 (Figure 4(B)). This interaction is not feasible, when the hydroxyl is switched to the R2 position to produce the otherwise identical (but inactive) analogue **9** (Figure 2). Moreover, the C2-carbonyl and the heterocyclic oxygen in the coumarin can H-bond with the Tyr219^OH^ in this “non-canonical” pose. Due to the flip, the Glu283^COO-^ cannot H-bond with **1** (Figure 4); however, the inward pose of the residue is not required for binding (PDB: 3KLM^45^). In this “non-canonical” pose, the R5-methoxy H-bonds with the Ser143^OH^ and, additionally, the Val144^N^ and Tyr156^OH^ are favourably oriented towards the polar group (Figure 4(B)).

### Cross-reactivity of the 3-phenylcoumarin analogues

It is not enough that drug candidates bind into their target proteins to elicit desired effects *in situ*. One also needs to consider their absorption, distribution, metabolism, and excretion (ADME) properties, toxicity, off-target effects, and overall selectivity. For example coumarins are known to produce hepatotoxic effects with a certain subgroup of humans – a phenomenon likely emerging from problems in the 7-hydroxylation of coumarins by the genetically polymorphic CYP2A6 enzyme^46^^,^^47^. Although no animal testing was performed in this study, the cross-reactivity of the 3-phenylcoumarin analogues was tested against ER, HSD2, CYP1A2, MAO-A, MAO-B, and aromatase using *in vitro* assays (Table 1).

Oestrogen receptor (ER) antagonists/agonists or selective oestrogen receptor modulators, such as tamoxifen and raloxifene are used routinely in treatment against ER-positive breast cancer. Potent HSD1 inhibitors could have a dual function as ER antagonists but they should not have a dual role as ER agonists promoting breast tissue tumourigenesis. Thus, the effect of the HSD1 inhibitor analogues was studied against both ER and of the potent HSD1 inhibitor analogues, only **5** was found to produce moderate ER inhibition. Of the more modest HSD1 inhibitor analogues **6** yielded reasonable ER inhibition. In addition, compounds **8** and **9**, which do not inhibit HSD1 activity, inhibited ER. The molecular basis for this is clear based on a prior study with the ER-specific compounds^14^: the 3-phenylcoumarin scaffold must have R2-functional group, e.g. R2-hydroxyl moiety, at its coumarin ring system to produce the inhibitory effect.

17-β-hydroxysteroid dehydrogenase 2 (HSD2), which is the enzymatic counterpart of HSD1, converts E_2_ to E_1_. Accordingly, to avoid counterproductive effects, it is paramount that any potential drugs aiming to lower the E_2_ production should not effectively block the HSD2 activity as a side effect. The activity testing indicates that none of the 3-phenylcoumarin analogues produce >50% HSD2 inhibition at 1 µM as the inhibition remains at a range from 7 to 42% (Table 1). Notably, the most potent HSD1 inhibitor analogues block the HSD2 only at a moderate level (**1** at 27%, **2** at 7%, and **4** at 16%; Table 1). If concentrating on the HSD2 activity, **2** is the most selective HSD1 inhibitor analogue while **4** is a close runner-up. Although **4** is the more potent HSD1 inhibitor of the two (or of all the tested analogues), the close to optimal H-bonding coordination with the R1- and R5-hydroxyls of **2** inside the HSD1 active site (Figure 3(A)) could be the underlying reason for its higher selectivity. However, the lack of 3D structural data on HSD2 or its homologous proteins, especially regarding the enzyme’s binding site, make it difficult to resolve this issue.

Monoamine oxidases (MAO) A and B are inhibited to some degree by the 3-phenylcoumarin analogues and this effect is notable for the MAO-B (see e.g.^18^^,^^48^^,^^49^). For that reason, the inhibition levels of the analogues were studied here against both enzyme subtypes (Table 1). Analogous to earlier studies^18^^,^^49–51^ analogues showing HSD1 inhibition also blocked the MAO-B activity at 10 µM. However, of the HSD1 inhibitor analogues presented in this study, only **6** (Figure 6(A)) and **7** have pIC_50_ above 6 (IC_50_ < 1 µM). Based on the docking, the R6-fluorine and R5-hydroxyl of **6** form a halogen bond and an H-bond with the Leu164^O^ and the Pro102^O^, respectively. Interestingly, **4** has been tested with MAO-A and MAO-B earlier (C6 in^18^). Although **4** reached 64% inhibition at 10 µM in our studies, it has shown more promising activity in a study by Delogu et al.^18^. Overall, the results suggest that the MAO-B inhibition would not be a critical issue for the new analogues or at least for the most potent of them.

Cytochrome P450 (CYP) enzymes metabolise majority of oestrogens first in the liver. In this vital process, CYP1A2 enzyme has a prominent role^52^ and, therefore, its unintended inhibition by a small-molecule could promote upswing in the effective E_2_ levels. Because the ultimate goal of any HSD1 inhibitor, including the 3-phenylcoumarins presented in this study, is to lower the E_2_ levels *in vivo*, their ability to block the CYP1A2 was studied as well. All of the analogues block CYP1A2 activity at some concentration, however, only the most potent HSD1 inhibitor **4** blocks its function at an alarming level (Table 1). The ligands that bind into the narrow and hydrophobic active site of CYP1A2 can be either substrates that are metabolised by the enzyme or inhibitors that block its function. As the substrates can be metabolised at different positions, it is unpractical to offer just one binding pose for each analogue. Regardless, for example the binding pose of **4**, which is the strongest CYP1A2 inhibitor of the analogue set (Table 1), likely reminds the validated pose of α-naphthoflavone (Figure 6(B))^28^. Based on the docking, the R1-chlorine of **4** packs against the haeme and the 3-phenyl ring is sandwiched between the side chains of Phe226 and Phe260 (not shown). Moreover, the C2-carbonyl of **4** forms an H-bond with a crystal water the same way as is seen for α-naphthoflavone and the Thr118^OH^ accepts an H-bond from the R4-hydroxyl (Figure 6(B)).

Aromatase (CYP19A1) inhibitors are used in breast cancer treatments, but unlike in the case of ER, their potential ability to bind into both HSD1 and aromatase could not be harmful. Aromatase inhibitors are predominantly used with post-menopausal breast cancer patients, because the E_2_ production via the aromatase pathway happens locally rather than relying on the ovaries^53^. In contrast, although the 3-phenylcoumarin scaffold mimics the steroidal core, and fit into the active site of the aromatase, analogues **1**–**9** do not produce aromatase inhibition (Table 1). A closer inspection indicates that this lack of activity is due to the inability of the polar R1–R6 groups to produce favourable interactions at the aromatase’s active site. On the one hand, the R1-positioned chlorine (**4**; Figure 2), methoxy (**7**; Figure 2), or hydroxyl group (**2**; Figure 2) could bond with the proton of Met374^N^. On the other hand, while the R4-hydroxyl groups of **2**, **3** and **4** are within the H-bonding range from the Asp309 side chain, this key residue is in a neutral state at pH 7.4 (PDB: 3EQM)^26^ and, therefore, ready to donate a proton instead of accepting one.

### 3-Imidazolecoumarin inhibits aromatase potently

The analysis of analogues **1**–**9** (see above) indicated that the coumarin-based compounds with flat ring systems at the 3-position could fit into the active site of the aromatase. However, a simple H-bond acceptor at the R4 position, such as a carbonyl group (of androstenedione in Figure 6(C)) would be needed to avoid the detrimental clash of proton donors between the bound ligand and the neutral Asp309 side chain at the active site. Instead of trying to “mutate” 3-phenylcoumarin core further to facilitate aromatase inhibition, a new kind of coumarin-derivative **10**, in which the 3-phenyl ring is substituted with a 3-imidazole was synthesised (Figure 2).

There are two potential binding poses at the aromatase’s active site for **10**. First, the deprotonated N3′ of the 3-imidazole ring could accept an H-bond from the neutral Asp309 side chain (Figure 6(C)). Second, the N3′ could coordinate directly with the haeme. Although the latter option was not put forward by the docking (not shown), the imidazole group is known to bind strongly with the haeme groups and induced-fit effects could help to accommodate it at the site. Nevertheless, the activity testing shows that **10** inhibits strongly the aromatase (pIC_50_ = 7.11; Table 1).

Furthermore, cross-reactivity testing of **10** indicates that the compound is blocking neither HSD1 nor MAO-B but it has a stronger inhibitory effect with CYP1A2 than with any of the 3-phenylcoumarin analogues (Table 1). The coumarin ring of **10** is likely to be flipped in a reverse pose inside the active site of CYP1A2 in comparison to the 3-phenylcoumarin analogues (Figure 6(B,D)). Importantly, in this pose the deprotonated and electronegative N3’ of imidazole would be coordinated with the positively charged iron in the middle of the haeme; meanwhile, the C2-carbonyl of **10** accepts an H-bond from the Thr124^OH^ (Figure 6(D)).

### 3-Phenylcoumarins are not pan-assay interference compounds

The cross-reactivity data demonstrates that coumarin with C3-substituted phenyl or imidazole ring does not belong to the pan assay interference compounds (PAINS) category, but that it is a privileged structure, which can be fine-tuned or tailored to function selectively with various targets. The PAINS filtering^54^, performed using CANVAS module of MAESTRO, supported this conclusion (no compounds filtered out).

Coumarins are a widely studied group of compounds with structural and pharmacological variability. Thus, it is not surprising that also 3-phenylcoumarins have been studied against other targets elsewhere. Some of the compounds published here have been previously tested for inhibitory activity against for HIV-1 replication (**1**; C17 in^15^), immune complex-mediated neutrophil oxidative metabolism (**2**; CHEMBL486894; C13 in^16^), glyceraldehyde-3-phosphate dehydrogenase (**3**; CHEMBL71407; C18 in^17^), and MAO-A and -B (**4**; C6 in^18^). All these compounds showed moderate ability to inhibit their intended targets. This further shows that 3-phenylcoumarins have interesting pharmacologic properties and that they have a broad utilisation range over therapeutic target proteins.

## Conclusions

3-Phenylcoumarin (Figure 2) is established here as a non-steroidal scaffold for building potent small-molecule HSD1 inhibitors. The 3-phenyl and coumarin rings are suggested to adopt similar hydrophobic packing at the active site as the established steroidal compounds (Figure 1(D)). Five of the 3-phenylcoumarin analogues produced ≥62% HSD1 inhibition at 5 µM (Table 1). Moreover, three of the analogues produced C68% inhibition even at 1 µM (**1**, **2**, and **4**; Figure 2; Table 1). The approximated pIC_50_ value at 1 µM for the three best analogues was ≥6.2. Housing polar moieties at the R5 and/or R6 positions in the 3-phenyl ring is generally critical for establishing the 3-phenylcoumarin binding and inhibition with HSD1 (Figure 5; Table 1). Introducing yet another polar group at the R1 position (Figure 5) in the coumarin ring boosts the HSD1 inhibition even further (e.g. **4**; Figure 3(E); Table 1). Moreover, inserting a hydroxyl group at the R3 position is expected to reverse the 3-phenylcoumarin binding at the active site (Figure 5) in comparison to the other analogues (**1**; Figures 3 and 4(B)) but without doing away with the inhibition (Table 1). A thorough cross-reactivity analysis highlights the fact that the 3-phenylcoumarin analogues block HSD2 only at moderate levels (Table 1), which is an essential feature for any potential drug candidates aiming to combat the E_2_-linked diseases, such as breast cancer and endometriosis. In addition, substituting the 3-phenyl with an imidazole changed the scaffold selectivity completely as the resulting compound **10** blocked potently the aromatase instead of the HSD1. To sum up, the coumarin core can be tailored to block the E_2_ synthesis by either the sulphatase pathway or the aromatase pathway by adding either a 3-phenyl or a 3-imidazole ring, respectively.
